# A Periodic Event-Triggered Design of Robust 
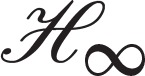
 Filtering for T-S Fuzzy Discrete-Time Systems

**DOI:** 10.3389/fnins.2019.00318

**Published:** 2019-04-17

**Authors:** Zuchang Zhang, Dongliang Lin, Xingyi Wang, Zhenhua Shao, Wenzhong Lin

**Affiliations:** ^1^Fujian Provincial Key Laboratory of Information Processing and Intelligent Control, Minjiang University, Fuzhou, China; ^2^Digital Fujian IoT Laboratory of Intelligent Production, Minjiang University, Fuzhou, China

**Keywords:** discrete-time Takagi-Sugeno (T-S) fuzzy systems, H_∞_ filtering, periodic event-triggered, robust control, perturbed and piecewise linear system approach

## Abstract

Periodic event-triggered control (PETC) is a control strategy consisting of event-triggered control (ETC) and conventional periodic sampled-data control. By using event-triggering mechanisms (ETM) to periodically verify whether or not to transmit and compute the measured output, communication and computational datum are significantly reduced while still retaining a satisfactory performance. This paper investigates the PETC scheme of robust H_∞_ filtering for a class of uncertain discrete-time Takagi-Sugeno (T-S) fuzzy systems, where the sample time is assumed to be a constant. To analyze the filtering problems of the PETC strategy, we present two frameworks based on perturbed linear and piecewise linear systems, to model filtering error systems. Sufficient conditions for the existence of a robust H_∞_ filter are derived in the form of matrix inequalities (LMIs) under these two frameworks, respectively. Finally, a simulation example is used to testify to the effectiveness of the proposed approach.

## 1. Introduction

T-S fuzzy models use a set of IF-THEN fuzzy rules to approximate complex nonlinear systems in terms of a set of local linear models that are connected smoothly by fuzzy membership functions at any preciseness (Sugeno, 1985; Tanaka and Wang, 2001). In other words, it can combine the merits of both the fuzzy logic theory and the linear system theory, and brings a 2-fold advantage: (i) any nonlinear systems can be approximately represented by the fuzzy dynamic models; (ii) the controller itself can be designed by utilizing the concept of parallel distributed compensation (PDC). Since a set of local linear models with T-S fuzzy rules can be used to represent a nonlinear system, it is a natural approach to design a local controller for each local model, respectively. In addition, digital fuzzy logic controllers (FLC) are successfully implemented in embedded microprocessors because of the availability of low-cost and high-speed computers, and are widely applied in a variety of engineering fields. Consequently, it becomes important to study problems of control for T-S fuzzy discrete-time systems, and there are some results on T-S fuzzy discrete-time systems in the open literature (Gao et al., 2005; Feng, 2006; Qiu et al., 2009; Wu et al., 2011; Zhong et al., 2013, 2019).

In many digital implementations of control systems, the embedded microprocessors forming the computational core of the control system are required to execute a variety of tasks, which consist of sampling the output of the plant, computing the input of the controller, and implementing the output of the controller. Under the execution of control tasks, two main schemes exist: time-triggered control and event-triggered control. The event-triggered control decides whether or not to execute the control task in terms of a given threshold, rather than a time-triggered control, in which the control task is carried out in a periodic manner. When compared to time-triggered control, the advantages of event-triggered control are 2-fold: a reduction in the data transmission and the computational cost of the controller. Over the past few years, there has been an increasing interest in event-triggered control, (see Hristu-Varsakelis and Kumar, 2002; Tabuada and Wang, 2006; Wang and Lemmon, 2011; Heemels and Donkers, 2013; Zhong and Zhu, 2017; Zhong, 2018, and references therein). The scheme of event-based control has appeared under several names, such as and periodic event-triggered control (PETC) (Heemels and Donkers, 2013; Zhong and Zhu, 2017), event-triggered feedback (Zhong, 2018), interrupt-based feedback Hristu-Varsakelis and Kumar (2002), self-triggered feedback Wang and Lemmon (2011), and state-triggered feedback Tabuada and Wang (2006).

Recently, an event-triggered scheme was studied for T-S fuzzy systems, and some results were reported in Guan et al. (2013); Jia et al. (2014), and He et al. (2013). It was noted that the controller designed in He et al. (2013) is based on the assumption that the premise variables between the fuzzy systems and the fuzzy controller are synchronous all the time. Alternatively, the condition that the premise variables between the fuzzy system and the controller are asynchronous is considered in Guan et al. (2013), Jia et al. (2014). In fact, it is worth pointing out that the premise variables of the controller decide whether or not to update, under the event-triggered strategy. In other words, if the difference between the current measured output and the most recently transmitted output value exceeds a specified threshold, then the premise variables of the controller are updated to the premise variables of the system. In this way, both the premise variables between the fuzzy system and the controller are synchronous. If the triggered condition is not satisfied, they are asynchronous. More recently, the premise variables with the PETC scheme was considered in Zhong and Zhu (2017, 2018), Zhong et al. (2018). More specifically, the work of Zhong et al. (2018) proposed a decentralized event-triggered mechanism for a class of large-scale networked fuzzy systems. The work in Zhong and Zhu (2018) introduced the asynchronous distributed event-triggered output-feedback controller to stabilize large-scale fuzzy systems. A distributed event-triggered controller was designed in Zhong and Zhu (2017) under a two-channel network.

On the other hand, the Kalman and H_∞_ filtering are the two main approaches among various filtering schemes. The H_∞_ filtering method minimizes the signal estimation error for the bounded disturbances and noise of the worst case, and does not require the exact knowledge of the statistics of the external noise signals. These two advantages render the H_∞_ filtering method very appropriate to practical applications. Most recently, some researchers have paid attention to state estimation/filtering problems for nonlinear systems (Yin et al., 2016, 2018; Lin et al., 2017; Yin and Liu, 2017; Zhu et al., 2018a,b). More specifically, the work of Yin and Liu (2017) focused on the distributed moving horizon estimation (DMHE) for a class of two-time-scale nonlinear systems described in the framework of singularly perturbed systems. The work in Yin et al. (2018) designed a distributed estimator for linear systems, deployed over sensor networks within a multiple communication channels (MCCs) framework. The HMM-based H_∞_ filtering problem for discrete-time markov jump LPV systems was studied via unreliable communication channels Zhu et al. (2018b). State and input simultaneous estimation for discrete-time switched singular delay systems were investigated under the missing measurements Lin et al. (2017). The H_∞_ estimation for a class of networked non-linear systems was considered in Yin et al. (2016). The problem of stability and stabilization for discrete-time switched PWA systems was studied by using a descriptor system approach in Zhu et al. (2018a).

Moreover, to the authors' best knowledge, few attempts have been researched on the H_∞_ filtering of T-S fuzzy systems under a PETC strategy, and the H_∞_ filtering of T-S fuzzy systems in the PETC strategy still remain open, which has motivated us to conduct this study.

In this paper, we will study the robust H_∞_ filtering design for a class of uncertain discrete-time T-S fuzzy systems under a PETC communication scheme, which is introduced to reduce the systematic resource, while preserving the desired performance. In this PETC scheme, the sample time is assumed to be a constant, and the measurement output and the premise variables of the filter are verified periodically on whether or not to update. Two frameworks based on perturbed linear and piecewise linear systems are presented to model the filtering error systems, respectively. By introducing a fuzzy-basis-dependent Lyapunov functional combined with Finsler lemma, sufficient conditions for the robust filtering PETC design of these two frameworks are derived, while satisfying a given H_∞_ performance index, and the filter gains can be obtained by solving a set of LMIs. Finally, an example is exploited in order to illustrate the effectiveness of the proposed results.

There are two main contributions in this paper. (i) Based on a PETC scheme, we study the robust H_∞_ filtering design for a class of uncertain discrete-time T-S fuzzy systems. To the best of our knowledge, relatively few theoretical results exist that study the PETC problem of robust H_∞_ filtering design for uncertain T-S fuzzy systems. (ii) In previous work on PETC approaches, the controller gains must be given a priori. In this paper, based on a fuzzy-basis-dependent Lyapunov functional and Finsler's lemma, the fuzzy H_∞_ filters for the filtering error systems, applying perturbed linear (PL) and the piecewise linear (PWL) system approaches, can be obtained by solving a set of linear matrix inequalities (LMIs).

**Notations**. The notations used throughout this paper are standard. ℝ^*n*^ and ℝ^*n*×*m*^ represent the *n*-dimensional Euclidean space and *n* × *m* real matrices. For a vector *x* ∈ ℝ^*n*^, we denote by $\left\|x\right\|:\;=\sqrt{x^{\text{T}}x}$ its 2-norm. The notation *P* > 0(≥ 0) means that the matrix *P* is positive (semi-positive) definite. For a matrix *A* ∈ ℝ^*n*×*n*^, *A*^−1^ and *A*^T^ are the inverse and transpose of the matrix *A*, respectively, and *A*^−T^ denotes (*A*^−1^)^T^. Sym{*A*} is the shorthand notation for *A*+*A*^T^. *I*_*n*_ denotes an identity matrix with dimension *n*. The symbol “^*^” in a matrix stands for the transposed elements in the symmetric positions. *l*_2_[0, ∞) refers to the space of square-integrable vector functions over [0, ∞). If not explicitly stated, matrices are assumed to have compatible dimensions for algebraic operations.

## 2. Model Description and Problem Formulation

Similar to Sugeno (1985), Tanaka and Wang (2001), Gao et al. (2005), Qiu et al. (2009), and Wu et al. (2011), a discrete-time T-S fuzzy dynamic model with parametric uncertainties can be described as follows:

***Plant Rule*** *R*^*i*^: **IF** ζ_1_(*k*) is $F_{1}^{i}$ and ζ_2_(*k*) is $F_{2}^{i}$ and … and ζ_*g*_(*k*) is $F_{g}^{i}$, **THEN**

(1) $$\left\{ \begin{array}{l} x\left(k+1\right)=\left(A_{i}+\Delta A_{i}\right)x\left(k\right)+\left(B_{i}+\Delta B_{i}\right)\omega \left(k\right),\\ y\left(k\right)=\left(C_{i}+\Delta C_{i}\right)x\left(k\right)+\left(D_{i}+\Delta D_{i}\right)\omega \left(k\right),\\ z\left(k\right)=\left(L_{i}+\Delta L_{i}\right)x\left(k\right)+\left(F_{i}+\Delta F_{i}\right)\omega \left(k\right),\;i\in L:=\left\{1,2\ldots ,r\right\}, \end{array}\right.$$

where *R*^*i*^ denotes the *i*th fuzzy inference rule, *r* is the number of inference rules, $\;F_{j}^{i}$ (*j* = 1, 2, ···, *g*) are fuzzy sets, $x\left(k\right)\in {\mathbb{R}}^{n_{x}}$ denotes the system state, $\omega \left(k\right)\in {\mathbb{R}}^{n_{\omega }}$ is the bounded external disturbance, $y\left(k\right)\in {\mathbb{R}}^{n_{y}}$ is the measurement output, $z\left(k\right)\in {\mathbb{R}}^{n_{z}}$ is the signal to be regulated, ζ(*k*): = [ζ_1_(*k*), ζ_2_(*k*), ⋯ζ_*g*_(*k*)] are some measurable variables of the system, (*A*_*i*_, *B*_*i*_, *C*_*i*_, *D*_*i*_, *L*_*i*_, *F*_*i*_) denotes the *i*th local model of the system, (Δ*A*_*i*_, Δ*B*_*i*_, Δ*C*_*i*_, Δ*D*_*i*_, Δ*L*_*i*_, Δ*F*_*i*_) denotes the uncertainty terms of the *i*th local model in the form of

(2) $$\left[\begin{array}{l} \Delta A_{i}\;\Delta B_{i}\\ \Delta C_{i}\;\Delta D_{i}\\ \Delta L_{i}\;\Delta F_{i} \end{array}\right]=\left[\begin{array}{l} M_{1i}\\ M_{2i}\\ M_{3i} \end{array}\right]\;\Delta \left(k\right)\left[N_{1i}\;N_{2i}\right],i\in L,$$

where *M*_1*i*_, *M*_2*i*_, *M*_3*i*_, *N*_1*i*_, and *N*_2*i*_ are known matrices, and $\Delta \left(k\right)\in {\mathbb{R}}^{n_{s_{2}\times s_{1}}}$ denotes the unknown time-varying matrix satisfying

(3) $$\Delta^{\text{T}}(k)\Delta (k)\leq I_{s_{1}}.$$

Let *h*_*i*_[ζ(*k*)] be the normalized fuzzy-basic-dependent function of the inferred fuzzy set F^*i*^, where F^*i*^:$=\prod_{\phi =1}^{\phi }$$F_{\phi }^{i}$, and

(4) $$h_{i}\left[\zeta (k)\right]:=\frac{\prod_{\phi =1}^{\phi }u_{i\phi }(\zeta_{\phi }(k))}{\sum_{\varsigma =1}^{r}\prod_{\phi =1}^{\phi }u_{\varsigma \phi }(\zeta_{\phi }(k))}\;\geq 0,\sum_{i=1}^{r}h_{i}\left[\zeta (k)\right]=1,$$

where *u*_*iϕ*_(ζ_ϕ_(*k*)) is the grade of membership of ζ_ϕ_(*k*) in $F_{\phi }^{i}.$

By using a center-average defuzzifier, product fuzzy inference, and a singleton fuzzifier, the following global T-S fuzzy dynamic model can be obtained:

(5) $$\{\begin{array}{l} x\left(k+1\right)=\left(A_{i}+\Delta A_{i}\right)x\left(k\right)+\left(B_{i}+\Delta B_{i}\right)\omega \left(k\right),\\ y\left(k\right)=\left(C_{i}+\Delta C_{i}\right)x\left(k\right)+\left(D_{i}+\Delta D_{i}\right)\omega \left(k\right),\\ z\left(k\right)=\left(L_{i}+\Delta L_{i}\right)x\left(k\right)+\left({ℱ}_{i}+\Delta {ℱ}_{i}\right)\omega \left(k\right), \end{array}$$

where

(6) $$\left[\begin{array}{cccc} A_{i} & \Delta A_{i} & B_{i} & \Delta B_{i}\\ C_{i} & \Delta C_{i} & D_{i} & \Delta D_{i}\\ L_{i} & \Delta L_{i} & {ℱ}_{i} & \Delta {ℱ}_{i} \end{array}\right]=\sum_{i=1}^{r}h_{i}\left[\zeta (k)\right]\left[\begin{array}{cccc} A_{i} & \Delta A_{i} & B_{i} & \Delta B_{i}\\ C_{i} & \Delta C_{i} & D_{i} & \Delta D_{i}\\ L_{i} & \Delta L_{i} & F_{i} & \Delta F_{i} \end{array}\right].$$

### 2.1. A PETC Strategy

For the filtering design, the traditional approach continually executing the filtering task may be undesirable in many situations. It leads to a conservative design (over-provisioning of the system hardware). In order to reduce the unnecessary waste of resources, we consider the PETC strategy outlined below:

(7) $$\hat{y}\left(k\right)=\{\begin{array}{ll} y\left(k\right)\text{,} & \text{when }\left\|y\left(k\right)-\hat{y}\left(k-1\right)\right\|>\delta \left\|y\left(k\right)\right\|,\\ \hat{y}\left(k-1\right)\text{,} & \text{when }\left\|y\left(k\right)-\hat{y}\left(k-1\right)\right\|\leq \delta \left\|y\left(k\right)\right\|, \end{array}$$

where ŷ(*k*) denotes the measurement output transmitting into the filtering system, δ ≥ 0 is a suitably chosen design parameter.

**Figure 1:**
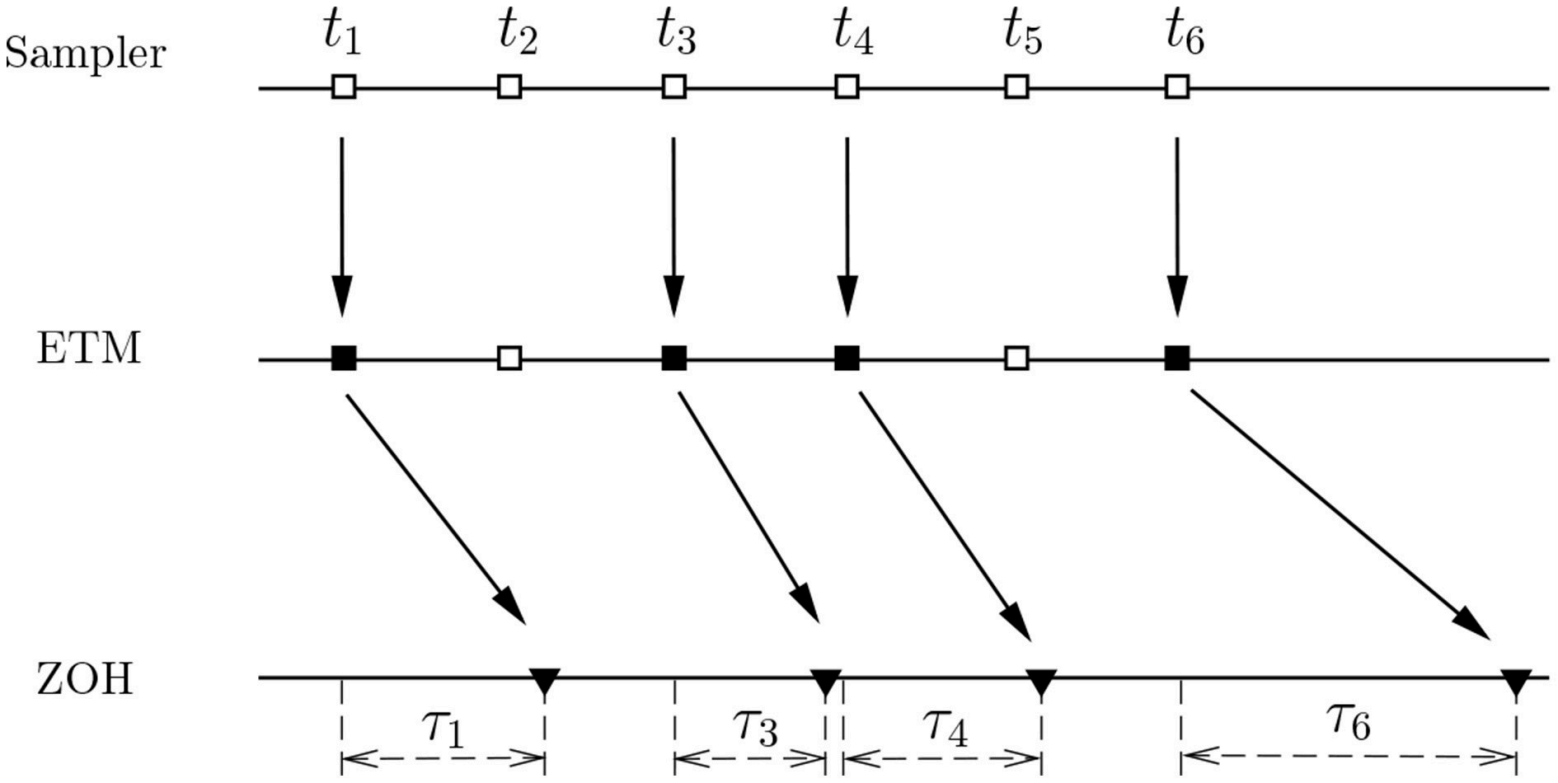
A PETC strategy with ETM in filtering system.

**Figure 2:**
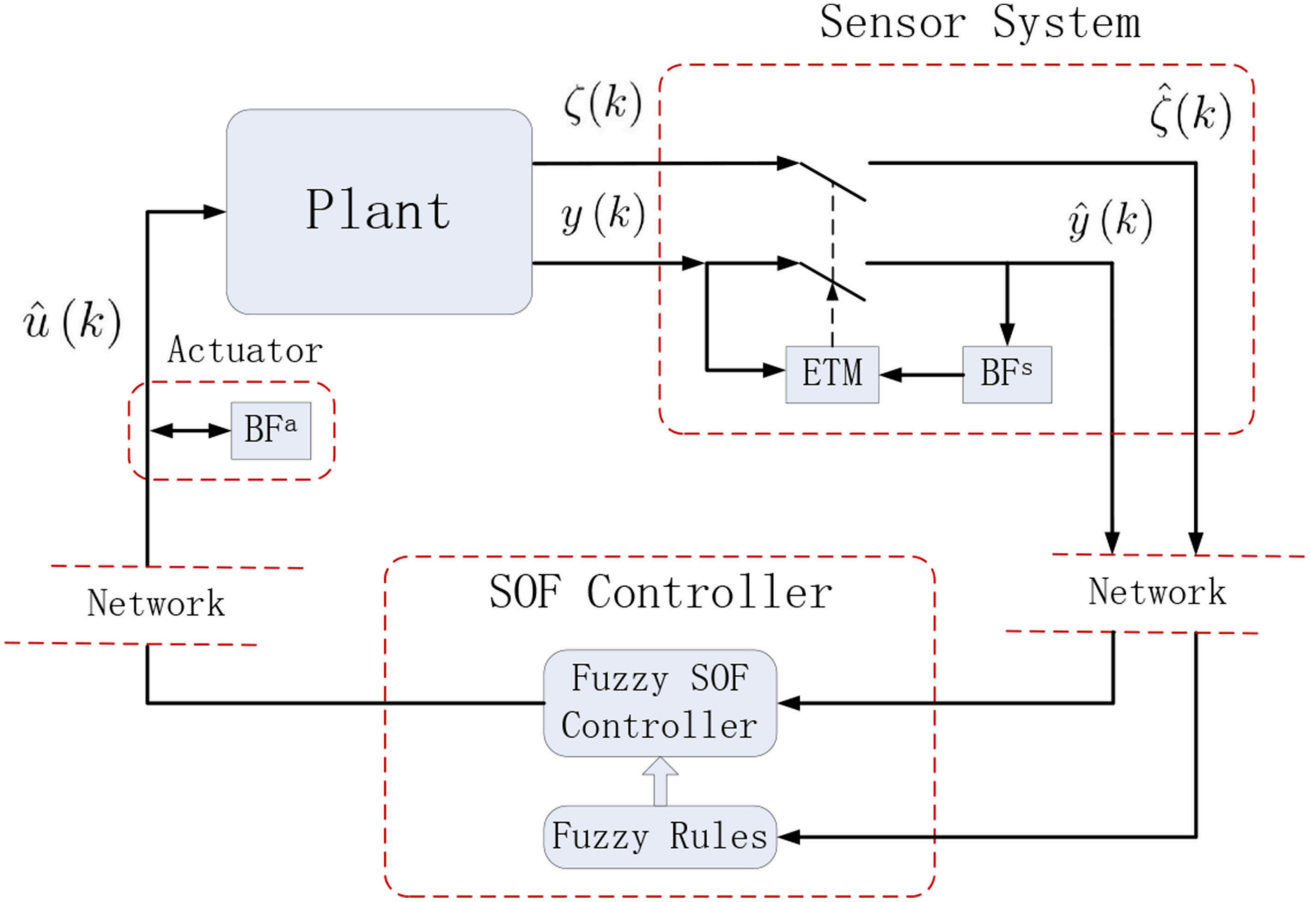
A PETC strategy with ETM in sensor system.

In practical implementations of the PETC strategy (7), we propose two different cases, see Figures 1, 2.

In Figure 1, two buffers located in the filtering system save the last measurement output transmitted to the filter and the last estimated signal, respectively. In every sample period, the event-triggering mechanism (ETM), based on the difference between the new measurement output and the last measurement output reserved in the buffer, determines whether or not to update ŷ(*k*). Both ŷ(*k*) and the fuzzy premise variables of the filter are updated when the difference exceeds a preselected threshold, and ŷ (*k*) will be executed by the filtering system. If not, the last estimated signal reserved in the other buffer is transmitted again and no date is executed in the filtering system. It should be noted that in this PETC strategy, the filter, the ETM, the buffers, and the fuzzy rule generator are designed in the filtering system, avoiding a long communication burden among them for the implementation of the filtering system. Thus, the PETC strategy is easy to implement in an inexpensive manner. However, it is impossible to reduce the transmitted datum. We will next present another PETC strategy which ensures a significant reduction in the number of transmission.

In this solution, a smart sensor system is proposed in Figure 2. The sensor system consists of a buffer stored at the last measurement output, and an ETM that determines whether or not to transmit the measurement output to the filtering system. The other buffer is to save the last estimated signal. Therefore, in every sample period the measurement output is transmitted to the filter and is executed only when the difference between the newly measurement output and the last measurement output transmitted to the filtering system exceeds a given threshold. Otherwise, the last estimated signal reserved in the other buffer will be transmitted again. In this way, this PETC strategy is capable of significantly reducing the number of filtering task transmission and executions, leading to a high cost and placing a long communication burden on the filter, the ETM, the buffers, and the fuzzy rule generator. This PETC strategy is also described by (7).

Now, given the fuzzy system (1) and the PETM strategy (7), a fuzzy filter for the estimation of *z*(*k*) with the structure described by

(8) $$\{\begin{array}{l} x_{F}\left(k+1\right)=A_{Fs}x_{F}\left(k\right)+B_{Fs}\hat{y}(k),\\ z_{F}\left(k\right)=C_{Fs}x_{F}\left(k\right)+D_{Fs}\hat{y}(k), \end{array}$$

where *x*_*F*_ (*k*) $\in {\mathbb{R}}^{n_{f}}$ is the filter state, *n*_*f*_ denotes the order of the fuzzy filter (*n*_*f*_ = *n*_*x*_ for the full-order filter and 1 ≤ *n*_*f*_ < *n*_*x*_ for the reduced-order filter), $z_{F}\left(k\right)\in {\mathbb{R}}^{n_{z}}$ an estimation of *z*(*k*), and *A*_*Fi*_, *B*_*Fi*_, *C*_*Fi*_, and *D*_*Fi*_ are appropriately dimensioned filter gains with the following form:

(9) $$\{\begin{array}{l} \left[\begin{array}{cc} A_{Fs} & B_{Fs}\\ C_{Fs} & D_{Fs} \end{array}\right]=\sum_{i=1}^{r}{\hat{h}}_{s}[\hat{\zeta }(k)]\left[\begin{array}{cc} A_{Fs} & B_{Fs}\\ C_{Fs} & D_{Fs} \end{array}\right],\\ {\hat{h}}_{s}\left[\hat{\zeta }(k)\right]:=\frac{\prod_{\phi =1}^{\phi }u_{s\phi }(\zeta_{\phi }(k))}{\sum_{\varsigma =1}^{r}\prod_{\phi =1}^{\phi }u_{\varsigma \phi }(\zeta_{\phi }(k))}\geq 0,\sum_{i=1}^{r}{\hat{h}}_{s}\left[\hat{\zeta }(k)\right]=1, \end{array}$$

and

(10) $$\hat{\zeta }(k)=\{\begin{array}{ll} \zeta (k)\text{,} & \text{when }\left\|y\left(k\right)-\hat{y}\left(k-1\right)\right\|>\delta \left\|y\left(k\right)\right\|,\\ \hat{\zeta }(k-1)\text{,} & \text{when }\left\|y\left(k\right)-\hat{y}\left(k-1\right)\right\|\leq \delta \left\|y\left(k\right)\right\|. \end{array}$$

For convenience, define

(11) $$h_{i}=h_{i}\left[\zeta (k)\right],h_{i}^{+}=h_{i}\left[\zeta (k+1)\right],{\hat{h}}_{i}={\hat{h}}_{i}[\hat{\zeta }(k)],i\in L.$$

**Remark 1**. It should be noted that the synchronous premise variables between T-S fuzzy systems and controllers are considered in He et al. (2013), and the asynchronous ones are considered in Guan et al. (2013), Jia et al. (2014). In fact, for the PECT strategy (7), the premise variables of the filter (8) are determined to decide whether or not to update by an ETM. In other words, the premise variables of the filter (8) are updated to the premise variables of the fuzzy system (5) when the newly measured output is transmitted to the filter. In this way, the premise variables between the filter (8) and the fuzzy system (5) are synchronous. Otherwise, they become asynchronous.

### 2.2. Closed-Loop System

#### 2.2.1. Perturbed Linear System

In order to apply a perturbed linear system approach proposed in Heemels et al. (2013), Zhong and Zhu (2017), we define

(12) $$e\left(k\right)=\hat{y}\left(k\right)-y\left(k\right),$$

and based on (7), yields

(13) 0≤e(k)≤δ‖y(k)‖,

where δ is a positive scalar.

By defining $\bar{x}(k)={\left[x^{\text{T}}(k)x_{F}^{\text{T}}(k)\right]}^{\text{T}},\bar{z}(k)=z(k)-z_{F}(k),$ and augmenting the model (5) and the filter (8), together with the consideration of (12), we obtained the filtering error system:

(14) $$\{\begin{array}{l} \bar{x}\left(k+1\right)=\left({\bar{A}}_{is}+\Delta {\bar{A}}_{is}\right)\bar{x}\left(k\right)+\left({\bar{B}}_{is}+\Delta {\bar{B}}_{is}\right)\omega \left(k\right)+{\bar{D}}_{is}e\left(k\right),\\ \bar{z}\left(k\right)=\left({\bar{C}}_{is}+\Delta {\bar{C}}_{is}\right)\bar{x}\left(k\right)+\left({\bar{ℰ}}_{is}+\Delta {\bar{ℰ}}_{is}\right)\omega \left(k\right)-D_{Fs}e\left(k\right), \end{array}$$

where

(15) $$\{\begin{array}{l} {\bar{A}}_{is}=\left[\begin{array}{cc} A_{i} & 0\\ B_{Fs}C_{i} & A_{Fs} \end{array}\right],\Delta {\bar{A}}_{is}=\left[\begin{array}{cc} \Delta A_{i} & 0\\ B_{Fs}\Delta C_{i} & 0 \end{array}\right],\\ {\bar{B}}_{is}=\left[\begin{array}{c} B_{i}\\ B_{Fs}D_{i} \end{array}\right],\Delta {\bar{B}}_{is}=\left[\begin{array}{c} \Delta B_{i}\\ B_{Fs}\Delta D_{i} \end{array}\right],{\bar{D}}_{is}=\left[\begin{array}{c} 0\\ B_{Fs} \end{array}\right],\\ {\bar{C}}_{is}=\left[\begin{array}{cc} L_{i}-D_{Fs}C_{i} & -C_{Fs} \end{array}\right],\Delta {\bar{C}}_{is}=\left[\begin{array}{cc} \Delta L_{i}-D_{Fs}\Delta C_{i} & 0 \end{array}\right],\\ {\bar{ℰ}}_{is}={ℱ}_{i}-D_{Fs}D_{i},\Delta {\bar{ℰ}}_{is}=\Delta {ℱ}_{i}-D_{Fs}\Delta D_{i}. \end{array}$$

#### 2.2.2. Piecewise Linear System

By defining $\tilde{x}(k)={\left[x^{\text{T}}(k)x_{F}^{\text{T}}(k){\hat{y}}^{\text{T}}(k-1)\right]}^{\text{T}}$, and augmenting the model (5) and the filter (8), we obtain the following closed-loop system:

(16) $$\{\begin{array}{l} \tilde{x}\left(k+1\right)=\left({\tilde{A}}_{is}^{1}+\Delta {\tilde{A}}_{is}^{1}\right)\tilde{x}\left(k\right)+\left({\tilde{B}}_{is}^{1}+\Delta {\tilde{B}}_{is}^{1}\right)\omega \left(k\right),\\ \bar{z}\left(k\right)=\left({\tilde{C}}_{is}^{1}+\Delta {\tilde{C}}_{is}^{1}\right)\bar{x}\left(k\right)+\left({\tilde{ℰ}}_{is}^{1}+\Delta {\tilde{ℰ}}_{is}^{1}\right)\omega \left(k\right), \end{array}$$

for ∥*y* (*k*) − ŷ (*k* − 1) ∥ > δ ∥ *y* (*k*) ∥, and

(17) $$\{\begin{array}{l} \tilde{x}\left(k+1\right)=\left({\tilde{A}}_{is}^{2}+\Delta {\tilde{A}}_{is}^{2}\right)\tilde{x}\left(k\right)+\left({\tilde{B}}_{is}^{2}+\Delta {\tilde{B}}_{is}^{2}\right)\omega \left(k\right),\\ \bar{z}\left(k\right)=\left({\tilde{C}}_{is}^{2}+\Delta {\tilde{C}}_{is}^{2}\right)\bar{x}\left(k\right)+\left({ℱ}_{i}+\Delta {ℱ}_{i}\right)\omega \left(k\right), \end{array}$$

for ∥*y* (*k*) − ŷ (*k* − 1) ∥ > δ ∥ *y* (*k*) ∥, and

(18) $$\{\begin{array}{l} {\tilde{A}}_{is}^{1}=\left[\begin{array}{ccc} A_{i} & 0 & 0\\ B_{Fs}C_{i} & A_{Fs} & 0\\ C_{i} & 0 & 0 \end{array}\right],{\tilde{B}}_{is}^{1}=\left[\begin{array}{c} B_{i}\\ B_{Fs}D_{i}\\ D_{i} \end{array}\right],\\ \Delta {\tilde{A}}_{is}^{1}=\left[\begin{array}{ccc} \Delta A_{i} & 0 & 0\\ B_{Fs}\Delta C_{i} & 0 & 0\\ \Delta C_{i} & 0 & 0 \end{array}\right],\Delta {\tilde{B}}_{is}^{1}=\left[\begin{array}{c} \Delta B_{i}\\ B_{Fs}\Delta D_{i}\\ \Delta D_{i} \end{array}\right],\\ {\tilde{C}}_{is}^{1}=\left[\begin{array}{ccc} L_{i}-D_{Fs}C_{i} & -C_{Fs} & 0 \end{array}\right],\Delta {\tilde{C}}_{is}^{1}=\left[\begin{array}{ccc} \Delta L_{i}-D_{Fs}\Delta C_{i} & 0 & 0 \end{array}\right],\\ {\tilde{ℰ}}_{is}^{1}={ℱ}_{i}-D_{Fs}D_{i},\Delta {\tilde{ℰ}}_{is}^{1}=\Delta {ℱ}_{i}-D_{Fs}\Delta D_{i}, \end{array}$$

and

(19) $$\{\begin{array}{l} {\tilde{A}}_{is}^{2}=\left[\begin{array}{ccc} A_{i} & 0 & 0\\ 0 & A_{Fs} & B_{Fs}\\ 0 & 0 & I \end{array}\right],{\tilde{B}}_{is}^{2}=\left[\begin{array}{c} B_{i}\\ 0\\ 0 \end{array}\right],\\ \Delta {\tilde{A}}_{is}^{2}=\left[\begin{array}{ccc} \Delta A_{i} & 0 & 0\\ 0 & 0 & 0\\ 0 & 0 & 0 \end{array}\right],\Delta {\tilde{B}}_{is}^{2}=\left[\begin{array}{c} \Delta B_{i}\\ 0\\ 0 \end{array}\right],\\ {\tilde{C}}_{is}^{2}=\left[\begin{array}{ccc} L_{i} & -C_{Fs} & -D_{Fs} \end{array}\right],\Delta {\tilde{C}}_{is}^{2}=\left[\begin{array}{ccc} \Delta L_{i} & 0 & 0 \end{array}\right]. \end{array}$$

Then the robust H_∞_ filtering design problem with the PETC strategy (7) is stated as follows:

Given the fuzzy discrete-time system (5), a fuzzy PETC filter (8) is designed to satisfy the following two requirement simultaneously:

**(a)** The filtering error system (14) based on the PL system approach (the filtering error system (16) and (17) based on the PWL system approach) with ω(*k*) = 0 is asymptotically stable;

**(b)** The induced *l*_2_ norm of the operator from ω to the filtering error $\bar{z}$ is less than γ under zero initial conditions

(20) $$H_{\infty }^{\bar{z}\omega }:=\sup\frac{{\left\|\bar{z}\right\|}_{2}}{{\left\|\omega \right\|}_{2}}<\gamma ,$$

for any nonzero ω ∈ *l*_2_[0 ∞) and all admissible uncertainties.

Before ending this section, the following lemmas are introduced to prove our main results.

**Lemma 1**. Xie (1996) Given constant matrices **X**, **Y**, and **Z** with **X** = **X**^T^ and 0 < **Y** = **Y**^T^, then **X** + **Z**^T^**Y**^−1^**Z** < 0 if and only if

$$\begin{array}{l} \left[\begin{array}{cc} \text{X} & {\text{Z}}^{\text{T}}\\ \text{Z} & -\text{Y} \end{array}\right]<0\text{ or }\left[\begin{array}{cc} -\text{Y} & \text{Z}\\ {\text{Z}}^{\text{T}} & \text{X} \end{array}\right]<0. \end{array}$$

**Lemma 2**. Xie (1996) Suppose that Δ(*k*) is given by (2) and (3), with matrices *M* = *M*^T^ and *S* and *N* of appropriate dimensions; then, the inequality

$$\begin{array}{l} M+\text{Sym}\left\{S\Delta \left(k\right)N\right\}<0, \end{array}$$

holds if, and only if, for some positive scalar ϵ > 0

$$\begin{array}{l} M+\left[\begin{array}{cc} \epsilon^{-1}N^{\text{T}} & \epsilon S \end{array}\right]\left[\begin{array}{cc} I & -J\\ -J^{\text{T}} & I \end{array}\right]\left[\begin{array}{c} \epsilon^{-1}N\\ \epsilon S^{\text{T}} \end{array}\right]<0. \end{array}$$

**Lemma 3**. de Oliveira and Skelton (2001) Let *x* ∈ ℝ^*n*^, ℙ = ℙ^T^ ∈ ℝ^*n*×*n*^, and ℍ ∈ ℝ^*m*×*n*^ such that rank (ℍ) = *r* < *n*. The following statements are equivalent:

1. 𝕏^T^ℙ𝕏 < 0 ∀ℍ𝕏 = 0, 𝕏 ≠ 0
2. (ℍ^⊥^)^T^ℙ(ℍ^⊥^) < 0,
3. ∃ℕ ∈ ℝ^*n*×*m*^:ℙ+ Sym{ℕℍ} < 0,
4. ∃λ ∈ ℝ:ℙ−λℍ^T^ℍ < 0.

## 3. Robust H_∞_ Filtering Analysis and Design

This section will carry out the filtering analysis and design with the PETC strategies by using the PL and PWL system approaches.

### 3.1. Perturbed Linear System

**Theorem 1**. Consider the fuzzy system (1) and the fuzzy filter (8) with the PETC strategy (7), the filtering error system (14) is robust asymptotically stabilization with H_∞_ performance γ if there exist sets of matrices $0<P_{i}=P_{i}^{\text{T}}\in \;{\mathbb{R}}^{\left(n_{x}+n_{f}\right)\times \left(n_{x}+n_{f}\right)},i\;\in \;L,G_{1s}\in {\mathbb{R}}^{n_{x}\times n_{x}},G_{2s}\;\in {\mathbb{R}}^{n_{f}\times n_{f}},G_{3s}\;\in \;{\mathbb{R}}^{n_{f}\times n_{x}},G_{4s}\;\in \;{\mathbb{R}}^{n_{z}\times n_{x}},G_{5s}\in {\mathbb{R}}^{n_{z}\times n_{z}},U_{i}\in {\mathbb{R}}^{\left(n_{x}+n_{f}+n_{w}\right)\times n_{x}},{Ā}_{Fs}\in {\mathbb{R}}^{n_{f}\times n_{f}},{\bar{B}}_{Fs}\in {\mathbb{R}}^{n_{f}\times n_{y}},{\bar{C}}_{Fs}\in {\mathbb{R}}^{n_{z}\times n_{f}},{\bar{D}}_{Fs}\in {\mathbb{R}}^{n_{z}\times n_{y}},s\in L,$ and some positive scalars δ, ε_*isj*_, (*i, s, j*) ∈ L, such that for all *j* ∈ L, the following LMIs hold

(21) $$\left[\begin{array}{ccc} \Sigma_{iij} & \varepsilon_{iij}E_{i}^{\text{T}} & W_{ii}\\ {}* & -\varepsilon_{iij}I_{s_{1}} & 0\\ {}* & * & -\varepsilon_{iij}I_{s_{1}} \end{array}\right]<0,i\in L,$$

and

(22) $$\left[\begin{array}{ccccc} \Sigma_{isj}+\Sigma_{sij} & \varepsilon_{isj}E_{i}^{\text{T}} & W_{is} & \varepsilon_{isj}E_{s}^{\text{T}} & W_{si}\\ {}* & -\varepsilon_{isj}I_{s_{1}} & 0 & 0 & 0\\ {}* & * & -\varepsilon_{isj}I_{s_{1}} & 0 & 0\\ {}* & * & * & -\varepsilon_{isj}I_{s_{1}} & 0\\ {}* & * & * & * & -\varepsilon_{isj}I_{s_{1}} \end{array}\right]<0,1\leq i<s\leq r,$$

where

(23) $$\left\{ \begin{array}{l} \Sigma_{isj}=\left[\begin{array}{cc} \Theta_{ij}+\text{Sym}\{\Xi_{is}\} & \delta \Pi_{i}^{\text{T}}\\ {}* & -I_{n_{y}} \end{array}\right],\\ \Pi_{i}=\left[\begin{array}{ccccc} 0_{n_{y}\times (n_{x}+n_{f}+n_{z})} & C_{i} & 0_{n_{y}\times n_{f}} & D_{i} & 0_{n_{y}\times n_{y}} \end{array}\right],\\ \Xi_{is}=\left[\begin{array}{ccccccc} -G_{1s} & -KG_{2s} & 0 & \Xi_{is}^{14} & K{\bar{A}}_{Fs} & \Xi_{is}^{16} & K{\bar{B}}_{Fs}\\ -G_{3s} & -G_{2s} & 0 & \Xi_{is}^{24} & {\bar{A}}_{Fs} & \Xi_{is}^{26} & {\bar{B}}_{Fs}\\ -G_{4s} & 0 & -G_{5s} & \Xi_{is}^{34} & -{\bar{C}}_{Fs} & \Xi_{is}^{36} & -{\bar{D}}_{Fs}\\ -U_{s} & 0 & 0 & U_{s}A_{i} & 0 & U_{s}B_{i} & 0 \end{array}\right],\\ \Xi_{is}^{14}=G_{1s}A_{i}+K{\bar{B}}_{Fs}C_{i},\Xi_{is}^{16}=G_{1s}B_{i}+K{\bar{B}}_{Fs}D_{i},\\ \Xi_{is}^{24}=G_{3s}A_{i}+{\bar{B}}_{Fs}C_{i},\Xi_{is}^{26}=G_{3s}B_{i}+{\bar{B}}_{Fs}D_{i},\\ \Xi_{is}^{34}=G_{4s}A_{i}+G_{5s}L_{i}-{\bar{D}}_{Fs}C_{i},\Xi_{is}^{36}=G_{4s}B_{i}+G_{5s}F_{i}-{\bar{D}}_{Fs}D_{i},\\ W_{is}=\left[\begin{array}{c} G_{1s}M_{1i}+K{\bar{B}}_{Fs}M_{2i}\\ G_{3s}M_{1i}+{\bar{B}}_{Fs}M_{2i}\\ G_{4s}M_{1i}+G_{5s}M_{3i}-{\bar{D}}_{Fs}M_{2i}\\ U_{s}M_{1i}\\ M_{2i} \end{array}\right],K={\left[\begin{array}{cc} I_{n_{f}} & 0_{n_{f}\times (n_{x}-n_{f})} \end{array}\right]}^{\text{T}},\\ E_{i}=\left[\begin{array}{ccccc} 0_{s_{1}\times (n_{x}+n_{f}+n_{z})} & N_{1i} & 0_{n_{f}} & N_{2i} & 0_{n_{y}} \end{array}\right], \end{array}\right.$$

and Θ_*il*_ is defined in (36).

Moreover, the proposed fuzzy filter in the form of (8) is given by

(24) $$\begin{array}{l} A_{Fs}=G_{2s}^{-1}{\bar{A}}_{Fs},B_{Fs}=G_{2s}^{-1}{\bar{B}}_{Fs},C_{Fs}=G_{5s}^{-1}{\bar{C}}_{Fs},D_{Fs}\\ \;=G_{5s}^{-1}{\bar{D}}_{Fs},s\in L. \end{array}$$

**Proof**. Consider a fuzzy-basis-dependent Lyapunov functional (Zhong et al., 2015):

(25) $$V(k)={\bar{x}}^{\text{T}}(k)P_{i}\bar{x}(k),$$

where $P_{i}=P_{i}^{\text{T}}>0,P_{i}=\underset{i=1}{\overset{r}{\sum }}h_{i}P_{i}.$

Define Δ*V*(*k*) = *V*(*k*+1)−*V*(*k*), and along the trajectory of the PL closed-loop system (14), yields

(26) $$\Delta V(k)={\bar{x}}^{\text{T}}(k+1)P_{j}\bar{x}(k+1)-{\bar{x}}^{\text{T}}(k)P_{i}\bar{x}(k).$$

It is well known that under zero initial conditions for any nonzero ω∈*l*_2_[0 ∞) and all admissible uncertainties in the filtering error system in (14) is asymptotically stable with H_∞_ performance, if the following inequality satisfies

(27) $$\Delta V(k)+{\bar{z}}^{\text{T}}\left(k\right)\bar{z}\left(k\right)-\gamma^{2}\omega^{\text{T}}\left(k\right)\omega \left(k\right)<0.$$

To facilitate the filtering design for the system (14), it has from (13) that

(28) $$\Delta V(k)+{\bar{z}}^{\text{T}}\left(k\right)\bar{z}\left(k\right)-\gamma^{2}\omega^{\text{T}}\left(k\right)\omega \left(k\right)<e^{\text{T}}(k)e(k)-\delta^{2}y^{\text{T}}\left(k\right)y\left(k\right),$$

which implies (27).

Now, we directly specify the slack matrix variables $G_{s}$ with the following form:

(29) $$\begin{array}{l} G_{s}=\left[\begin{array}{ccc} G_{1s} & KG_{2s} & 0_{n_{x}\times n_{z}}\\ G_{3s} & G_{2s} & 0_{n_{f}\times n_{z}}\\ G_{4s} & 0_{n_{z}\times n_{f}} & G_{5s}\\ U_{s} & 0_{\left(n_{x}+n_{f}+n_{w}+n_{y}\right)\times n_{f}} & 0_{\left(n_{x}+n_{f}+n_{w}+n_{y}\right)\times n_{z}} \end{array}\right]\\ =\underset{s=1}{\sum^{r}}{\hat{h}}_{s}\left[\begin{array}{ccc} G_{1s} & KG_{2s} & 0_{n_{x}\times n_{z}}\\ G_{3s} & G_{2s} & 0_{n_{f}\times n_{z}}\\ G_{4s} & 0_{n_{z}\times n_{f}} & G_{5s}\\ U_{s} & 0_{\left(n_{x}+n_{f}+n_{w}+n_{y}\right)\times n_{f}} & 0_{\left(n_{x}+n_{f}+n_{w}+n_{y}\right)\times n_{z}} \end{array}\right]. \end{array}$$

In addition, it follows from (14) that

(30) $$A\left(i,s,\Delta \right)\xi_{1}\left(k\right)=0,$$

where

(31) $$\{\begin{array}{l} A\left(i,s,\Delta \right)=\left[\begin{array}{ccccc} -I_{\left(n_{x}+n_{f}\right)} & 0 & {\bar{A}}_{is}+\Delta {\bar{A}}_{is} & {\bar{B}}_{is}+\Delta {\bar{B}}_{is} & {\bar{D}}_{is}\\ 0 & -I_{n_{z}} & {\bar{C}}_{is}+\Delta {\bar{C}}_{is} & {\bar{ℰ}}_{is}+\Delta {\bar{ℰ}}_{is} & -{\bar{D}}_{Fis} \end{array}\right],\\ \xi_{1}(k)={\left[\begin{array}{ccccc} {\bar{x}}^{\text{T}}\left(k+1\right) & {\bar{z}}^{\text{T}}\left(k\right) & {\bar{x}}^{\text{T}}\left(k\right) & \omega^{\text{T}}\left(k\right) & e^{\text{T}}\left(k\right) \end{array}\right]}^{\text{T}},\xi_{1}(k)\neq 0. \end{array}$$

Then, based on Finsler's lemma (Lemma 3), substituting the matrix $G_{s}$ defined in (29) into (30), and together with (28), yields

(32) $$\xi_{1}^{\text{T}}(k)\left[\Theta (i,j)+\delta_{i}^{2}\Pi^{\text{T}}\left(i,\Delta \right)\Pi \left(i,\Delta \right)+\text{Sym}\{\Xi \left(i,s,\Delta \right)\}\right]\xi_{1}(k)<0,$$

where

(33) $$\left\{ \begin{array}{l} \Theta (i,j)=\left[\begin{array}{ccccc} P_{j} & 0 & 0 & 0 & 0\\ {}* & I_{n_{z}} & 0 & 0 & 0\\ {}* & 0 & -P_{i} & 0 & 0\\ {}* & * & * & -\gamma^{2}I_{n_{w}} & 0\\ {}* & * & * & * & -I_{n_{y}} \end{array}\right],\\ \Pi \left(i,\Delta \right)=\left[\begin{array}{ccccc} 0_{n_{y}\times (n_{x}+n_{f}+n_{z})} & C_{i}+\Delta C_{i} & 0_{n_{y}\times n_{f}} & D_{i}+\Delta D_{i} & 0_{n_{y}\times n_{y}} \end{array}\right],\\ \Xi \left(i,s,\Delta \right)=[\begin{array}{cccc} -G_{1s} & -KG_{2s} & 0 & \Xi^{14}\left(i,s,\Delta \right)\\ -G_{3s} & -G_{2s} & 0 & \Xi^{24}\left(i,s,\Delta \right)\\ -G_{4s} & 0 & -G_{5s} & \Xi^{34}\left(i,s,\Delta \right)\\ -U_{s} & 0 & 0 & U_{s}\left(A_{i}+\Delta A_{i}\right) \end{array}\\ \begin{array}{ccc} G_{2s}A_{Fs} & G_{1s}\left(B_{i}+\Delta B_{i}\right)+KG_{2s}B_{Fs}\left(D_{i}+\Delta D_{i}\right) & KG_{2s}B_{Fs}\\ G_{2s}A_{Fs} & G_{3s}\left(B_{i}+\Delta B_{i}\right)+G_{2s}B_{Fs}\left(D_{i}+\Delta D_{i}\right) & G_{2s}B_{Fs}\\ -G_{5s}C_{Fs} & \Xi^{36}\left(i,s,\Delta \right) & -G_{5s}D_{Fs}\\ 0 & U_{s}\left(B_{i}+\Delta B_{i}\right) & 0 \end{array}],\\ \Xi^{14}\left(i,s,\Delta \right)=G_{1s}\left(A_{i}+\Delta A_{i}\right)+KG_{2s}B_{Fs}\left(C_{i}+\Delta C_{i}\right),\\ \Xi^{24}\left(i,s,\Delta \right)=G_{3s}\left(A_{i}+\Delta A_{i}\right)+G_{2s}B_{Fs}\left(C_{i}+\Delta C_{i}\right),\\ \Xi^{34}\left(i,s,\Delta \right)=G_{4s}\left(A_{i}+\Delta A_{i}\right)+G_{5s}\left(L_{i}+\Delta L_{i}\right)-G_{5s}D_{Fs}\left(C_{i}+\Delta C_{i}\right),\\ \Xi^{36}\left(i,s,\Delta \right)=G_{4s}\left(B_{i}+\Delta B_{i}\right)+G_{5s}\left({ℱ}_{i}+\Delta {ℱ}_{i}\right)-G_{5s}D_{Fs}\left(D_{i}+\Delta D_{i}\right). \end{array}\right.$$

By applying the Schur complement lemma (Lemmas 1), it is clear that the following inequality implies (32):

(34) $$\Sigma (i,,s,l,\Delta )=\left[\begin{array}{cc} \Theta (i,l)+\text{Sym}\{\Xi \left(i,s,\Delta \right)\} & \delta \Pi^{\text{T}}\left(i,\Delta \right)\\ {}* & -I_{n_{y}} \end{array}\right]<0.$$

According to (6, 9, 11) and (15, 34) can be easily rewritten as

(35) $$\begin{array}{l} \Sigma (i,,s,j,\Delta )=\underset{j=1}{\sum^{r}}h_{j}^{+}\underset{i=1}{\sum^{r}}h_{i}{\hat{h}}_{i}\Sigma_{iij}(\Delta )+\underset{j=1}{\sum^{r}}h_{j}^{+}\underset{i=1}{\sum^{r-1}}\underset{s=i+1}{\sum^{r}}h_{i}{\hat{h}}_{s}\{\Sigma_{isj}(\Delta )\\ \;+\Sigma_{sij}(\Delta )\}<0, \end{array}$$

where

(36) $$\left\{ \begin{array}{l} \Sigma_{isj}(\Delta )=\left[\begin{array}{cc} \Theta_{ij}+\text{Sym}\{\Xi_{is}(\Delta )\} & \delta \Pi_{i}^{\text{T}}(\Delta )\\ {}* & -I_{n_{y}} \end{array}\right],\\ \Theta_{ij}=\left[\begin{array}{ccccc} P_{j} & 0 & 0 & 0 & 0\\ {}* & I_{n_{z}} & 0 & 0 & 0\\ {}* & * & -P_{i} & 0 & 0\\ {}* & * & * & -\gamma^{2}I_{n_{w}} & 0\\ {}* & * & * & * & 0_{n_{y}\times n_{y}} \end{array}\right],\\ \Pi_{i}(\Delta )=\left[\begin{array}{ccccc} 0_{n_{y}\times (n_{x}+n_{f}+n_{z})} & C_{i}+\Delta C_{i} & 0_{n_{y}\times n_{f}} & D_{i}+\Delta D_{i} & 0_{n_{y}\times n_{y}} \end{array}\right],\\ \Xi_{is}(\Delta )=[\begin{array}{cccc} -G_{1s} & -KG_{2s} & 0 & \Xi_{is}^{14}(\Delta )\\ -G_{3s} & -G_{2s} & 0 & \Xi_{is}^{24}(\Delta )\\ -G_{4s} & 0 & -G_{5s} & \Xi_{is}^{34}(\Delta )\\ -U_{s} & 0 & 0 & U_{s}\left(A_{i}+\Delta A_{i}\right) \end{array}\\ \begin{array}{ccc} KG_{2s}A_{Fs} & G_{1s}\left(B_{i}+\Delta B_{i}\right)+KG_{2s}B_{Fi}\left(D_{i}+\Delta D_{i}\right) & KG_{2i}B_{Fs}\\ G_{2s}A_{Fs} & G_{3i}\left(B_{i}+\Delta B_{i}\right)+G_{2i}B_{Fi}\left(D_{i}+\Delta D_{i}\right) & G_{2s}B_{Fs}\\ -G_{5s}C_{Fs} & \Xi_{is}^{36}(\Delta ) & -G_{5s}D_{Fs}\\ 0 & U_{s}\left(B_{i}+\Delta B_{i}\right) & 0 \end{array}],\\ \Xi_{is}^{14}(\Delta )=G_{1s}\left(A_{i}+\Delta A_{i}\right)+KG_{2s}B_{Fs}\left(C_{i}+\Delta C_{i}\right),\\ \Xi_{is}^{24}(\Delta )=G_{3s}\left(A_{i}+\Delta A_{i}\right)+G_{2s}B_{Fs}\left(C_{i}+\Delta C_{i}\right),\\ \Xi_{is}^{34}(\Delta )=G_{4s}\left(A_{i}+\Delta A_{i}\right)+G_{5s}\left(L_{i}+\Delta L_{i}\right)-G_{5s}D_{Fs}\left(C_{i}+\Delta C_{i}\right),\\ \Xi_{is}^{36}(\Delta )=G_{4s}\left(B_{i}+\Delta B_{i}\right)+G_{5s}\left(F_{i}+\Delta F_{i}\right)-G_{5s}D_{Fs}\left(D_{i}+\Delta D_{i}\right). \end{array}\right.$$

Since $h_{i}\left[\zeta \left(k\right)\right]\geq 0,\underset{l=1}{\overset{r}{\sum }}h_{i}\left[\zeta \left(k\right)\right]=1,$ and ${ĥ}_{s}\left[\hat{\zeta }\left(k\right)\right]\geq 0,\underset{s=1}{\overset{r}{\sum }}{ĥ}_{s}\left[\hat{\zeta }\left(k\right)\right]=1,$ so that the following inequalities imply (34):

(37) $$\begin{array}{ll} \Sigma_{iij}(\Delta )<0, & i\in L, \end{array}$$

and

(38) $$\begin{array}{ll} \Sigma_{isj}(\Delta )+\Sigma_{sij}(\Delta )<0, & 1\leq i<s\leq r. \end{array}$$

On the other hand, using relations (2–3), one has

(39) $$\Sigma_{isj}(\Delta )=\Sigma_{isj}+\text{Sym}\left\{W_{is}\Delta (k)E_{i}\right\},$$

where Σ_*isj*_, *W*_*is*_, and *E*_*i*_ are defined in (22).

By introducing

(40) $${\bar{A}}_{Fs}=G_{2s}A_{Fs},{\bar{B}}_{Fs}=G_{2s}B_{Fs},{\bar{C}}_{Fs}=G_{5s}C_{Fs},{\bar{D}}_{Fs}=G_{5s}D_{Fs},s\in L,$$

and by applying the Schur complement and S-procedure (Lemmas 1 and 2) to (43), it is clear that (25) and (26) are obtained, respectively. The proof is therefore completed.

### 3.2. Piecewise Linear System

Based on the PWL closed-loop system given by (16, 17), we will present the filtering design as follows:

**Theorem 2**. Given the fuzzy system (1), and an admissible fuzzy filter (8) with the PETC strategy (7), the filtering error system given by (16) and (17) is robust asymptotically, stabilized with H_∞_ performance γ if there exist sets of matrices $0<P_{i}^{m}={\left(P_{i}^{m}\right)}^{\text{T}}\in {\mathbb{R}}^{\left(n_{x}+n_{f}+n_{y}\right)\times \left(n_{x}+n_{f}+n_{y}\right)},$ $\left(i,m\right)\in L;Y_{11s}^{m}\in {\mathbb{R}}^{n_{x}\times n_{x}},Y_{2s}\in {\mathbb{R}}^{n_{f}\times n_{f}},Y_{13s}^{m}\in {\mathbb{R}}^{n_{x}\times n_{y}},Y_{21s}^{m}\in {\mathbb{R}}^{n_{f}\times n_{x}},Y_{23s}^{m}\in {\mathbb{R}}^{n_{f}\times n_{y}},Y_{31s}^{m}\in {\mathbb{R}}^{n_{z}\times n_{x}},$ $Y_{33s}^{m}\in {\mathbb{R}}^{n_{z}\times n_{y}},Y_{41s}^{m}\in {\mathbb{R}}^{n_{y}\times n_{x}},Y_{43s}^{m}\in {\mathbb{R}}^{n_{y}\times n_{y}},Y_{5s}\in {\mathbb{R}}^{n_{z}\times n_{z}},Q_{1i}^{m}\in {\mathbb{R}}^{\left(n_{x}+n_{f}+n_{y}+n_{w}\right)\times n_{x}},$ $Q_{2i}^{m}\in {\mathbb{R}}^{\left(n_{x}+n_{f}+n_{y}+n_{w}\right)\times n_{y}},{Ã}_{Fs}\in {\mathbb{R}}^{n_{f}\times n_{f}},{\tilde{B}}_{Fs}\in {\mathbb{R}}^{n_{f}\times n_{y}},{\tilde{C}}_{Fs}\in {\mathbb{R}}^{n_{z}\times n_{f}},{\tilde{D}}_{Fs}\in {\mathbb{R}}^{n_{z}\times n_{y}},\left(m,s\right)\in L;$ and some positive scalars δ, *k*_*m*_, ε_*isj*_, (*m, i, s, j*)∈L, such that for all *j* ∈ L, the following LMIs hold

(41) $$\left[\begin{array}{ccc} {\tilde{\Theta }}_{ij}^{nm}+\text{Sym}\left\{{\tilde{\Xi }}_{ii}^{m}\right\} & -\varepsilon_{iij}^{m}{\tilde{E}}_{i} & {\tilde{W}}_{ii}^{m}\\ {}* & -\varepsilon_{iij}^{m}I_{n_{s_{1}}} & 0\\ {}* & * & -\varepsilon_{iij}^{m}I_{n_{s_{1}}} \end{array}\right]<0,$$

for *i* ∈ L, (*n, m*) = {1, 2}, and

(42) $$\left[\begin{array}{ccccc} {\tilde{\Theta }}_{ij}^{nm}+{\tilde{\Theta }}_{ji}^{nm}+\text{Sym}\left\{{\tilde{\Xi }}_{is}^{m}+{\tilde{\Xi }}_{si}^{m}\right\} & -\varepsilon_{isj}^{m}{\tilde{E}}_{i} & {\tilde{W}}_{is}^{m} & -\varepsilon_{isj}^{m}{\tilde{E}}_{s} & {\tilde{W}}_{si}^{m}\\ {}* & -\varepsilon_{isj}^{m}I_{n_{s_{1}}} & 0 & 0 & 0\\ {}* & * & -\varepsilon_{isj}^{m}I_{n_{s_{1}}} & 0 & 0\\ {}* & * & * & -\varepsilon_{isj}^{m}I_{n_{s_{1}}} & 0\\ {}* & * & * & * & -\varepsilon_{isj}^{m}I_{n_{s_{1}}} \end{array}\right]<0,$$

for 1 ≤ *i* < *s* ≤ *r*, (*n, m*) = {1, 2}, and

(43) $$\{\begin{array}{l} {\tilde{\Theta }}_{ij}^{nm}=\left[\begin{array}{cccc} P_{j}^{n} & 0 & 0 & 0\\ {}* & I_{n_{z}} & 0 & 0\\ {}* & 0 & -P_{i}^{m} & 0\\ {}* & * & * & -\gamma^{2}I_{n_{w}} \end{array}\right],\\ {\tilde{E}}_{i}=\left[\begin{array}{ccccc} 0_{s_{1}\times (n_{x}+n_{f}+n_{y}+n_{z})} & N_{1i} & 0_{s_{1}\times n_{f}} & 0_{s_{1}\times n_{y}} & N_{2i} \end{array}\right], \end{array}$$

and

(44) $$\left\{ \begin{array}{l} {\tilde{\Xi }}_{is}^{1}=[\begin{array}{ccccccc} -Y_{11s}^{1} & -KY_{2s} & -Y_{13s}^{1} & 0 & {\tilde{\Xi }}_{15is}^{1} & K{\tilde{A}}_{Fs} & 0\\ -Y_{21s}^{1} & -Y_{2j} & -Y_{23s}^{1} & 0 & {\tilde{\Xi }}_{25is}^{1} & {\tilde{A}}_{Fs} & 0\\ -Y_{31s}^{1} & 0 & -Y_{33s}^{1} & 0 & Y_{31s}^{1}A_{i}+Y_{33s}^{1}C_{i} & 0 & 0\\ -Y_{41s}^{1} & 0 & -Y_{43s}^{1} & -Y_{5j} & {\tilde{\Xi }}_{45is}^{1} & -{\tilde{C}}_{Fs} & 0\\ -Q_{1s}^{1} & 0 & -Q_{2s}^{1} & 0 & Q_{1s}^{1}A_{i}+Q_{2s}^{1}C_{i} & 0 & 0 \end{array}\\ \begin{array}{c} {\tilde{\Xi }}_{18is}^{1}\\ {\tilde{\Xi }}_{28is}^{1}\\ Y_{31s}^{1}B_{i}+Y_{33s}^{1}D_{i}\\ {\tilde{\Xi }}_{48is}^{1}\\ Q_{1s}^{1}B_{i}+Q_{2s}^{1}D_{i} \end{array}],{\tilde{W}}_{is}^{1}=\left[\begin{array}{c} Y_{11s}^{1}M_{1i}+\left(KY_{2s}B_{Fs}+Y_{13s}^{1}\right)M_{2i}\\ Y_{21s}^{1}M_{1i}+\left(Y_{2s}B_{Fs}+Y_{23s}^{1}\right)M_{2i}\\ Y_{31s}^{1}M_{1i}+Y_{33s}^{1}M_{2i}\\ Y_{41s}^{1}M_{1i}+\left(Y_{43s}^{1}-{\tilde{D}}_{Fs}\right)M_{2i}+Y_{5s}M_{3i}\\ Q_{1s}^{1}M_{1i}+Q_{2s}^{1}M_{2i} \end{array}\right]\\ {\tilde{\Xi }}_{15is}^{1}=Y_{11s}^{1}A_{i}+K{\tilde{B}}_{FS}C_{i}+Y_{13s}^{1}C_{i},{\tilde{\Xi }}_{18is}^{1}=Y_{11s}^{1}B_{i}+K{\tilde{B}}_{FS}D_{i}+Y_{13s}^{1}D_{i},\\ {\tilde{\Xi }}_{25is}^{1}=Y_{21s}^{1}A_{i}+{\tilde{B}}_{FS}C_{i}+Y_{13s}^{1}C_{i},{\tilde{\Xi }}_{28is}^{1}=Y_{21s}^{1}B_{i}+{\tilde{B}}_{FS}D_{i}+Y_{23s}^{1}D_{i},\\ {\tilde{\Xi }}_{45is}^{1}=Y_{41s}^{1}A_{i}+Y_{43s}^{1}C_{i}+Y_{5s}L_{i}-{\tilde{D}}_{FS}C_{i},\\ {\tilde{\Xi }}_{48is}^{1}=Y_{41s}^{1}B_{i}+Y_{43s}^{1}D_{i}+Y_{5s}L_{Fi}-{\tilde{D}}_{FS}D_{i}, \end{array}\right.$$

and

(45) $$\{\begin{array}{l} {\tilde{\Xi }}_{is}^{2}=[\begin{array}{ccccc} -Y_{11s}^{2} & -KY_{2s} & -Y_{13s}^{2} & 0 & Y_{11s}^{2}A_{i}\\ -Y_{21s}^{2} & -Y_{2s} & -Y_{23s}^{2} & 0 & Y_{21s}^{2}A_{i}\\ -Y_{31s}^{2} & 0 & -Y_{33s}^{2} & 0 & Y_{31s}^{2}A_{i}\\ -Y_{41s}^{2} & 0 & -Y_{43s}^{2} & -Y_{5s} & Y_{41s}^{2}A_{i}+Y_{5s}L_{i}\\ Q_{1s}^{2} & 0 & Q_{2s}^{2} & 0 & Q_{1s}^{2}A_{i} \end{array}\\ \begin{array}{ccc} K{\tilde{A}}_{Fs} & K{\tilde{B}}_{Fs}+Y_{13s}^{2} & Y_{11s}^{2}B_{i}\\ K{\tilde{A}}_{Fs} & {\tilde{B}}_{Fs}+Y_{23s}^{2} & Y_{21s}^{2}B_{i}\\ 0 & Y_{23s}^{2} & Y_{31s}^{2}B_{i}\\ -{\tilde{C}}_{Fs} & Y_{43s}^{2}-{\tilde{D}}_{Fs} & Y_{41s}^{2}B_{i}+Y_{5s}F_{i}\\ 0 & Q_{2s}^{2} & Q_{1s}^{2}B_{i} \end{array}],{\tilde{W}}_{is}^{2}=\left[\begin{array}{c} Y_{11s}^{2}M_{1i}\\ Y_{21s}^{2}M_{1i}\\ Y_{31s}^{2}M_{1i}\\ Y_{41s}^{2}M_{1i}+Y_{5s}M_{3i}\\ Q_{1s}^{2}M_{1i} \end{array}\right]. \end{array}$$

Moreover, an admissible fuzzy filter in the form of (8) is given by

(46) $$\begin{array}{l} A_{Fs}=Y_{2s}^{-1}{\tilde{A}}_{Fs},B_{Fs}=Y_{2s}^{-1}{\tilde{B}}_{Fs},C_{Fs}=Y_{5s}^{-1}{\tilde{C}}_{Fs},D_{Fs}\\ \;=Y_{5s}^{-1}{\tilde{D}}_{Fs},s\in L. \end{array}$$

**Proof**. Consider a piecewise quadratic Lyapunov functional Zhong and Zhu (2018):

(47) $$V(k)={\bar{x}}^{\text{T}}\left(k\right)P_{i}\bar{x}\left(k\right),$$

where $P_{i}^{m}={\left(P_{i}^{m}\right)}^{\text{T}}>0,P_{i}^{m}=\sum_{i=1}^{r}h_{i}P_{i}^{m},m=\left\{1,2\right\}.$

Along the trajectory of two subsystems given by (16, 17), yields

(48) $$\Delta V(k)={\bar{x}}^{\text{T}}\left(k+1\right)P_{j}^{n}\bar{x}\left(k+1\right)-{\bar{x}}^{\text{T}}\left(k\right)P_{i}^{m}\bar{x}\left(k\right).$$

It follows from (7, 27), that

(49) $$\begin{array}{l} \Delta V(k)+{\bar{z}}^{\text{T}}\left(k\right)\bar{z}\left(k\right)-\gamma^{2}{\bar{\omega }}^{\text{T}}\left(k\right)\bar{\omega }\left(k\right)<{\left(-1\right)}^{m}\eta_{m}\\ \left\{{\left[y\left(k\right)-\hat{y}\left(k-1\right)\right]}^{\text{T}}(★)-\delta^{2}y^{\text{T}}\left(k\right)y\left(k\right)\right\}, \end{array}$$

where η_*m*_ ≥ 0, and the inequality (49) implies

(50) $$\Delta V(k)+{\bar{z}}^{\text{T}}\left(k\right)\bar{z}\left(k\right)-\gamma^{2}{\bar{\omega }}^{\text{T}}\left(k\right)\bar{\omega }\left(k\right)<0.$$

It is noted that the even-triggered information is particularly useful to reduce the conservatism of the systems with an event-triggered strategy. For the systems with parameter uncertainties involved in the matrices $C_{i}$ and $D_{i}$, the terms *y*^T^(*k*)*y*(*k*) and [*y*(*k*) − ŷ(*k* − 1)]^T^(⋆) can be separated by applying the Schur complement and S-procedure lemma. However, it is easy to see that it will lead to a infeasible solution in (49). As a result, (49) is only suitable for the case of the matrices $C_{i}$ and $D_{i}$ without uncertainties. Otherwise, (50) should be used.

Based on (50), and similar to (29–31), the following results can be obtained

(51) $$\xi_{2}^{\text{T}}(k)\left[\tilde{\Theta }(n,j,m,i)+\text{Sym}\{Y^{m}(s)A^{m}(i,s,\Delta )\}\right]\xi_{2}(k)<0,$$

where (*m, n*) = {1, 2}, and

(52) $$\left\{ \begin{array}{l} A^{1}(i,j,\Delta )=\left[\begin{array}{cccc} -I & 0 & {\tilde{A}}_{is}^{1}+\Delta {\tilde{A}}_{is}^{1} & {\tilde{B}}_{is}^{1}+\Delta {\tilde{B}}_{is}^{1}\\ 0 & -I & {\tilde{C}}_{is}^{1}+\Delta {\tilde{C}}_{is}^{1} & {\tilde{ℰ}}_{is}^{1}+\Delta {\tilde{ℰ}}_{is}^{1} \end{array}\right],\\ A^{2}(i,s,\Delta )=\left[\begin{array}{cccc} -I & 0 & {\tilde{A}}_{is}^{2}+\Delta {\tilde{A}}_{is}^{2} & {\tilde{B}}_{is}^{2}+\Delta {\tilde{B}}_{is}^{2}\\ 0 & -I & {\tilde{C}}_{is}^{2}+\Delta {\tilde{C}}_{is}^{2} & {ℱ}_{i}+\Delta {ℱ}_{i} \end{array}\right],\\ \tilde{\Theta }(n,j,m,i)=\left[\begin{array}{cccc} P_{j}^{n} & 0 & 0 & 0\\ {}* & I_{n_{z}} & 0 & 0\\ {}* & 0 & -P_{i}^{m} & 0\\ {}* & * & * & -\gamma^{2}I_{n_{w}} \end{array}\right],\\ \xi_{2}(k)={\left[\begin{array}{cccc} {\tilde{x}}^{\text{T}}\left(k+1\right) & {\bar{z}}^{\text{T}}\left(k\right) & {\tilde{x}}^{\text{T}}\left(k\right) & \omega^{\text{T}}\left(k\right) \end{array}\right]}^{\text{T}},\xi_{2}(k)\neq 0. \end{array}\right.$$

We directly specify the slack matrix variables:

(53) $$\begin{array}{l} Y^{m}(s)=\left[\begin{array}{cccc} Y_{11s}^{m} & KY_{2s} & Y_{13s}^{m} & 0_{n_{x}\times n_{z}}\\ Y_{21s}^{m} & Y_{2s} & Y_{23s}^{m} & 0_{n_{f}\times n_{z}}\\ Y_{31s}^{m} & 0_{n_{y}\times n_{f}} & Y_{33s}^{m} & 0_{n_{y}\times n_{z}}\\ Y_{41s}^{m} & 0_{n_{z}\times n_{f}} & Y_{43s}^{m} & Y_{5s}\\ Q_{1s}^{m} & 0_{\left(n_{x}+n_{f}+n_{y}+n_{w}\right)\times n_{f}} & Q_{2s}^{m} & 0_{\left(n_{x}+n_{f}+n_{y}+n_{w}\right)\times n_{z}} \end{array}\right]\\ =\underset{s=1}{\sum^{r}}{\hat{h}}_{s}\left[\begin{array}{cccc} Y_{11s}^{m} & KY_{2s} & Y_{13s}^{m} & 0_{n_{x}\times n_{z}}\\ Y_{21s}^{m} & Y_{2s} & Y_{23s}^{m} & 0_{n_{f}\times n_{z}}\\ Y_{31s}^{m} & 0_{n_{y}\times n_{f}} & Y_{33s}^{m} & 0_{n_{y}\times n_{z}}\\ Y_{41s}^{m} & 0_{n_{z}\times n_{f}} & Y_{43s}^{m} & Y_{5s}\\ Q_{1s}^{m} & 0_{\left(n_{x}+n_{f}+n_{y}+n_{w}\right)\times n_{f}} & Q_{2s}^{m} & 0_{\left(n_{x}+n_{f}+n_{y}+n_{w}\right)\times n_{z}} \end{array}\right]. \end{array}$$

According to (2, 6, 9) and (24), the following inequality implies (12):

(54) $$\begin{array}{l} {\tilde{\Sigma }}^{nm}(i,s,j,\Delta )=\underset{j=1}{\sum^{r}}h_{j}^{+}\underset{i=1}{\sum^{r}}h_{i}{\hat{h}}_{i}{\tilde{\Sigma }}_{iij}^{nm}(\Delta )+\underset{j=1}{\sum^{r}}h_{j}^{+}\underset{i=1}{\sum^{r-1}}\underset{s=i+1}{\sum^{r}}h_{i}{\hat{h}}_{s}\\ \left\{{\tilde{\Sigma }}_{isj}^{nm}(\Delta )+{\tilde{\Sigma }}_{sij}^{nm}(\Delta )\right\}<0, \end{array}$$

where

(55) $${\tilde{\Sigma }}_{isj}^{m}(\Delta )={\tilde{\Theta }}_{ij}^{nm}+\text{Sym}\{{\tilde{\Xi }}_{is}^{m}(\Delta )\},{\tilde{\Theta }}_{ij}^{nm}=\left[\begin{array}{cccc} P_{j}^{n} & 0 & 0 & 0\\ {}* & I_{n_{z}} & 0 & 0\\ {}* & 0 & -P_{i}^{m} & 0\\ {}* & * & * & -\gamma^{2}I_{n_{w}} \end{array}\right],$$

and

(56) $$\left\{ \begin{array}{l} {\tilde{\Xi }}_{is}^{1}(\Delta )=[\begin{array}{ccccccc} -Y_{11s}^{1} & -KY_{2s} & -Y_{13s}^{1} & 0 & {\tilde{\Xi }}_{15is}^{1}(\Delta ) & KY_{2s}A_{Fs} & 0\\ -Y_{21s}^{1} & -Y_{2j} & -Y_{23s}^{1} & 0 & {\tilde{\Xi }}_{25is}^{1}(\Delta ) & Y_{2s}A_{Fs} & 0\\ -Y_{31s}^{1} & 0 & -Y_{33s}^{1} & 0 & {\tilde{\Xi }}_{35is}^{1}(\Delta ) & 0 & 0\\ -Y_{41s}^{1} & 0 & -Y_{43s}^{1} & -Y_{5j} & {\tilde{\Xi }}_{45is}^{1}(\Delta ) & -Y_{5s}C_{Fs} & 0\\ -Q_{1s}^{1} & 0 & -Q_{2s}^{1} & 0 & {\tilde{\Xi }}_{55is}^{1}(\Delta ) & 0 & 0 \end{array}\\ \begin{array}{c} Y_{11s}^{1}\left(B_{i}+\Delta B_{i}\right)+KY_{2s}B_{Fs}\left(D_{i}+\Delta D_{i}\right)+Y_{13s}^{1}\left(D_{i}+\Delta D_{i}\right)\\ Y_{21s}^{1}\left(B_{i}+\Delta B_{i}\right)+Y_{2s}B_{Fs}\left(D_{i}+\Delta D_{i}\right)+Y_{23s}^{1}\left(D_{i}+\Delta D_{i}\right)\\ Y_{31s}^{1}\left(B_{i}+\Delta B_{i}\right)+Y_{33s}^{1}\left(D_{i}+\Delta D_{i}\right)\\ {\tilde{\Xi }}_{48is}^{1}(\Delta )\\ Q_{1s}^{1}\left(B_{i}+\Delta B_{i}\right)+Q_{2s}^{1}\left(D_{i}+\Delta D_{i}\right) \end{array}],\\ {\tilde{\Xi }}_{15is}^{1}(\Delta )=Y_{11s}^{1}\left(A_{i}+\Delta A_{i}\right)+KY_{2s}B_{Fs}\left(C_{i}+\Delta C_{i}\right)+\\ \;+Y_{13s}^{1}\left(C_{i}+\Delta C_{i}\right),\\ {\tilde{\Xi }}_{25is}^{1}(\Delta )=Y_{21s}^{1}\left(A_{i}+\Delta A_{i}\right)+Y_{2s}B_{Fs}\left(C_{i}+\Delta C_{i}\right)\\ \;+Y_{23s}^{1}\left(C_{i}+\Delta C_{i}\right),\\ {\tilde{\Xi }}_{35is}^{1}(\Delta )=Y_{31s}^{1}\left(A_{i}+\Delta A_{i}\right)+Y_{33s}^{1}\left(C_{i}+\Delta C_{i}\right),\\ {\tilde{\Xi }}_{45is}^{1}(\Delta )=Y_{41s}^{1}\left(A_{i}+\Delta A_{i}\right)+Y_{43s}^{1}\left(C_{i}+\Delta C_{i}\right)\\ \;+Y_{5s}\left(L_{i}+\Delta L_{i}\right)-Y_{5s}D_{Fs}\left(C_{i}+\Delta C_{i}\right),\\ {\tilde{\Xi }}_{55is}^{1}(\Delta )=Q_{1s}^{1}\left(A_{i}+\Delta A_{i}\right)+Q_{2s}^{1}\left(C_{i}+\Delta C_{i}\right),\\ {\tilde{\Xi }}_{48is}^{1}(\Delta )=Y_{41s}^{1}\left(B_{i}+\Delta B_{i}\right)+Y_{43s}^{1}\left(D_{i}+\Delta D_{i}\right)\\ \;+Y_{5s}\left(F_{i}+\Delta F_{i}\right)-Y_{5s}D_{Fs}\left(D_{i}+\Delta D_{i}\right), \end{array}\right.$$

and

(57) $$\left\{ \begin{array}{l} {\tilde{\Xi }}_{is}^{2}(\Delta )=[\begin{array}{ccccc} -Y_{11s}^{2} & -KY_{2s} & -Y_{13s}^{2} & 0 & Y_{11s}^{2}\left(A_{i}+\Delta A_{i}\right)\\ -Y_{21s}^{2} & -Y_{2s} & -Y_{23s}^{2} & 0 & Y_{21s}^{2}\left(A_{i}+\Delta A_{i}\right)\\ -Y_{31s}^{2} & 0_{n_{f}\times n_{z}} & -Y_{33s}^{2} & 0 & Y_{31s}^{2}\left(A_{i}+\Delta A_{i}\right)\\ -Y_{41s}^{2} & 0_{n_{z}\times n_{f}} & -Y_{43s}^{2} & -Y_{5s} & {\tilde{\Xi }}_{45is}^{2}(\Delta )\\ Q_{1s}^{2} & 0_{\left(n_{x}+n_{f}+n_{w}+n_{y}\right)\times n_{f}} & Q_{2s}^{2} & 0 & Q_{1s}^{2}\left(A_{i}+\Delta A_{i}\right) \end{array}\\ \begin{array}{ccc} KY_{2s}A_{Fs} & KY_{2s}B_{Fs}+Y_{13s}^{2} & Y_{11s}^{2}\left(B_{i}+\Delta B_{i}\right)\\ KY_{2s}A_{Fs} & Y_{2s}B_{Fs}+Y_{23s}^{2} & Y_{21s}^{2}\left(B_{i}+\Delta B_{i}\right)\\ 0 & Y_{23s}^{2} & Y_{31s}^{2}\left(B_{i}+\Delta B_{i}\right)\\ -Y_{5s}C_{Fs} & Y_{43s}^{2}-Y_{5s}D_{Fs} & Y_{41s}^{2}\left(B_{i}+\Delta B_{i}\right)+Y_{5s}\left(F_{i}+\Delta F_{i}\right)\\ 0 & Q_{2s}^{2} & Q_{1s}^{2}\left(B_{i}+\Delta B_{i}\right) \end{array}],\\ {\tilde{\Xi }}_{45is}^{2}(\Delta )=Y_{41s}^{2}\left(A_{i}+\Delta A_{i}\right)+Y_{5s}\left(L_{i}+\Delta L_{i}\right)\text{.} \end{array}\right.$$

The following proof is similar to the proof of Theorem 1 and is, therefore, omitted.

It is worth pointing out that for the case of the matrices $C_{i}$ and $D_{i}$ without uncertainties, the event-triggered information can be used to reduce the conservatism. The corresponding H_∞_ filtering design result can be readily obtained from Theorem 2 by including the event-triggered information. The result is summarized in the following corollary.

**Corollary 3**. Consider the fuzzy system (1) and the fuzzy filter (8) with the PETC strategy (7), the filtering error system given by (16, 17) is robust asymptotically stabilized with H_∞_ performance γ if there exist sets of matrices $0<P_{i}^{m}={\left(P_{i}^{m}\right)}^{\text{T}}\in {\mathbb{R}}^{\left(n_{x}+n_{f}+n_{y}\right)\times \left(n_{x}+n_{f}+n_{y}\right)},$ $\left(i,m\right)\in L;Y_{11s}^{m}\in {\mathbb{R}}^{n_{x}\times n_{x}},Y_{2s}\in {\mathbb{R}}^{n_{f}\times n_{f}},Y_{13s}^{m}\in {\mathbb{R}}^{n_{x}\times n_{y}},Y_{21s}^{m}\in {\mathbb{R}}^{n_{f}\times n_{x}},Y_{23s}^{m}\in {\mathbb{R}}^{n_{f}\times n_{y}},Y_{31s}^{m}\in {\mathbb{R}}^{n_{z}\times n_{x}},$ $Y_{33s}^{m}\in {\mathbb{R}}^{n_{z}\times n_{y}},Y_{41s}^{m}\in {\mathbb{R}}^{n_{y}\times n_{x}},Y_{43s}^{m}\in {\mathbb{R}}^{n_{y}\times n_{y}},Y_{5s}\in {\mathbb{R}}^{n_{z}\times n_{z}},Q_{1i}^{m}\in {\mathbb{R}}^{\left(n_{x}+n_{f}+n_{y}+n_{w}\right)\times n_{x}},$ $Q_{2i}^{m}\in {\mathbb{R}}^{\left(n_{x}+n_{f}+n_{y}+n_{w}\right)\times n_{y}},{Ã}_{Fs}\in {\mathbb{R}}^{n_{f}\times n_{f}},{\tilde{B}}_{Fs}\in {\mathbb{R}}^{n_{f}\times n_{y}},{\tilde{C}}_{Fs}\in {\mathbb{R}}^{n_{z}\times n_{f}},{\tilde{D}}_{Fs}\in {\mathbb{R}}^{n_{z}\times n_{y}},\left(m,s\right)\in L;$ and some positive scalars δ, *k*_*m*_, ε_*isj*_, (*m, i, s, j*) ∈ L, such that for all *j* ∈ L, the following LMIs hold

(58) $$\left[\begin{array}{ccc} {\tilde{\Theta }}_{ij}^{nm}+\text{Sym}\left\{{\tilde{\Xi }}_{is}^{m}\right\} & -\varepsilon_{iij}^{m}{\tilde{E}}_{i}^{m} & {\tilde{W}}_{is}^{m}\\ {}* & -\varepsilon_{iij}^{m}I_{n_{s_{1}}} & 0\\ {}* & * & -\varepsilon_{iij}^{m}I_{n_{s_{1}}} \end{array}\right]<0,$$

for *i*∈L, (*n, m*) = {1, 2}, and

(59) $$\left[\begin{array}{ccccc} {\tilde{\Theta }}_{ij}^{nm}+{\tilde{\Theta }}_{sj}^{nm}+\text{Sym}\left\{{\tilde{\Xi }}_{is}^{m}+{\tilde{\Xi }}_{si}^{m}\right\} & -\varepsilon_{isj}^{m}{\tilde{E}}_{i}^{m} & {\tilde{W}}_{is}^{m} & -\varepsilon_{isj}^{m}{\tilde{E}}_{s}^{m} & {\tilde{W}}_{si}^{m}\\ {}* & -\varepsilon_{isj}^{m}I_{n_{s_{1}}} & 0 & 0 & 0\\ {}* & * & -\varepsilon_{isj}^{m}I_{n_{s_{1}}} & 0 & 0\\ {}* & * & * & -\varepsilon_{isj}^{m}I_{n_{s_{1}}} & 0\\ {}* & * & * & * & -\varepsilon_{isj}^{m}I_{n_{s_{1}}} \end{array}\right]<0,$$

for 1 ≤ *i*<*s* ≤ *r*, (*n, m*) = {1, 2}, and ${\tilde{\Xi }}_{is}^{m}$ and Ẽ_*i*_ are defined in (42), and

(60) $$\left\{ \begin{array}{l} {\tilde{\Theta }}_{ij}^{nm}=\left[\begin{array}{cccc} P_{j}^{n} & 0 & 0 & 0\\ {}* & I_{n_{z}} & 0 & 0\\ {}* & 0 & -P_{i}^{m} & 0\\ {}* & * & * & -\gamma^{2}I_{n_{w}} \end{array}\right]+{\left(-1\right)}^{m+1}\eta_{m}\left[\begin{array}{c} 0_{\left(n_{x}+n_{f}+n_{y}+n_{z}\right)\times n_{y}}\\ C_{i}^{\text{T}}\\ -I_{n_{y}}\\ D_{i}^{\text{T}} \end{array}\right]\left(★\right)\\ +{\left(-1\right)}^{m}\eta_{m}\left[\begin{array}{c} 0_{\left(n_{x}+n_{f}+n_{y}+n_{z}\right)\times n_{y}}\\ \delta C_{i}^{\text{T}}\\ 0_{n_{y}\times n_{y}}\\ \delta D_{i}^{\text{T}} \end{array}\right]\left(★\right),\\ W_{is}^{1}=\left[\begin{array}{c} Y_{11s}^{1}M_{1i}\\ Y_{21s}^{1}M_{1i}\\ Y_{31s}^{1}M_{1i}\\ Y_{41s}^{1}M_{1i}+Y_{5s}M_{3i}\\ Q_{1s}^{1}M_{1i} \end{array}\right],W_{is}^{2}=\left[\begin{array}{c} Y_{11s}^{2}M_{1i}\\ Y_{21s}^{2}M_{1i}\\ Y_{31s}^{2}M_{1i}\\ Y_{41s}^{2}M_{1i}+Y_{5s}M_{3i}\\ Q_{1s}^{2}M_{1i} \end{array}\right], \end{array}\right.$$

Moreover, an admissible fuzzy filter in the form of (8) is given by

(61) $$\begin{array}{l} A_{Fs}=Y_{2s}^{-1}{\tilde{A}}_{Fs},B_{Fs}=Y_{2s}^{-1}{\tilde{B}}_{Fs},C_{Fs}=Y_{5s}^{-1}{\tilde{C}}_{Fs},D_{Fs}\\ \;=Y_{5s}^{-1}{\tilde{D}}_{Fs},s\in L. \end{array}$$

Note that the H_∞_ performance index γ described in Theorem 1, 2, and Corollary 3 can be optimized by the following algorithms:

Algorithm 1: min γ, subject to LMIs (21)-(22),
Algorithm 2: min γ, subject to LMIs (41)-(42),
Algorithm 3: min γ, subject to LMIs (58)-(59).

## 4. Simulation Examples

This section uses an example to illustrate the effectiveness of the proposed PETC filter design approach.

Consider a tunnel diode circuit, whose modeling was done in Assawinchaichote and Nguang (2003), that is

(62) $$\{\begin{array}{l} C{\dot{x}}_{1}\left(t\right)=-0.002x_{1}\left(t\right)-0.01x_{1}^{3}\left(t\right)+x_{2}\left(t\right),\\ L{\dot{x}}_{2}\left(t\right)=-x_{1}\left(t\right)-Rx_{2}\left(t\right)+\omega \left(t\right),\\ y(k)=Sx\left(k\right)+\omega \left(k\right),\\ z\left(k\right)=x_{1}\left(t\right), \end{array}$$

where *x*_1_(*t*) and *x*_2_(*t*) are the state variables, ω(*t*) is the disturbance noise input, *y*(*t*) is the measurement, *z*(*t*) is the controlled output, *C, L, R*, and *S* are the capacitance, the inductance, the resistance, and *S* the sensor matrix, respectively.

Given *C* = 20*mF*, *L* = 1000*mH* and *R* = 10Ω, consider the uncertainty Δ*R* = 0.1Ω. With a sampling time T = 0.02, the nonlinear system (62) can be approximated by the following discrete-time T-S model:

***Plant Rule*** *R*^*i*^: **IF** *x*_1_(*k*) is *F*^*i*^, **THEN**

(63) $$\{\begin{array}{l} x\left(k+1\right)=\left(A_{i}+\Delta A\right)x\left(k\right)+B_{i}\omega \left(k\right),\\ y(k)=Cx\left(k\right)+D\omega \left(k\right),\\ z\left(k\right)=Lx\left(k\right),\;i=\left\{1,2\right\} \end{array}$$

where

$\begin{array}{l} A_{1}=\left[\begin{array}{cc} 0.9887 & 0.9024\\ -0.0180 & 0.8100 \end{array}\right],B_{1}=\left[\begin{array}{c} 0.0093\\ 0.0181 \end{array}\right],\\ A_{2}=\left[\begin{array}{cc} 0.9034 & 0.8617\\ -0.0172 & 0.8103 \end{array}\right],B_{2}=\left[\begin{array}{c} 0.0091\\ 0.0181 \end{array}\right],\\ \Delta A=\left[\begin{array}{cc} 0 & 0\\ 0 & -0.0016 \end{array}\right],C=\left[\begin{array}{cc} 1 & 0 \end{array}\right],D=1,L=\left[\begin{array}{cc} 1 & 0 \end{array}\right]. \end{array}$

The uncertainty Δ*A* is assumed to satisfy the form of (2, 3), that is

(64) ΔA=MΔ(k)N,

where Δ(*k*) = sin(*k*), *M* = $\left[\begin{array}{c} 0\\ -0.16 \end{array}\right],N=\left[\begin{array}{cc} 0 & 0.01 \end{array}\right].$

Similar to Gao et al. (2009), the membership functions are given by

(65) $$\begin{array}{l} h_{1}\;=\{\begin{array}{l} \frac{x_{1}(k)+3}{3},-3\leq x_{1}(k)\leq 0,\\ 0,x_{1}(k)<-3,\\ \frac{3-x_{1}(k)}{3},0\leq x_{1}(k)\leq 3,\\ 0,x_{1}(k)>3, \end{array}\\ h_{2}=1-h_{1}. \end{array}$$

Firstly, the objective here is to design the PETC fuzzy filter (8) without A task delay, such that the filtering error system (13) is asymptotically stable with robust H_∞_ performance γ_min_. Given δ = 0.2, and applying Algorithm 1, the filter matrices and the minimum H_∞_ performance γ_min_ are obtained as follows:


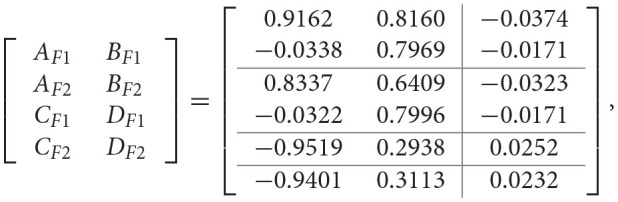


γ_min_ = 0.4,

for the full-order case, and


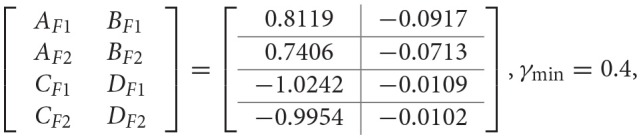


for the reduced-order (*n*_*f*_ = 1) case.

Then, we are in a position to design the PETC fuzzy filter (14) with a task delay such that the filtering error system (18) is asymptotically stable with the H_∞_ performance γ_min_. Given δ = 0.2, and the task delay *d*(*k*) is assumed to satisfy (15) with 0 ≤ *d*(*k*) ≤ 5, and by applying Algorithm 2, the filter matrices and the minimum H_∞_ performance γ_min_ are obtained as follows:


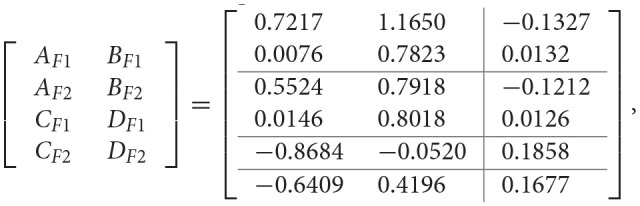


γ_min_ = 0.86.

However, by applying Algorithm 3, the filter matrices and the minimum H_∞_ performance γ_min_ are obtained as follows:


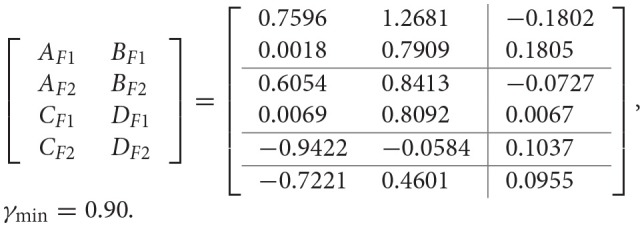


γ_min_ = 0.90.

## 5. Conclusion

In this paper, we have investigated the filtering design for a class of uncertain discrete-time T-S fuzzy systems under a periodic event-triggered control (PETC) scheme, where the sample time was assumed to be a constant. These two frameworks, based on perturbed and piecewise linear systems, were presented to model the filtering error systems, respectively. Based on a fuzzy-basis-dependent Lyapunov functional combined with Finsler's lemma, sufficient conditions for the robust filtering PETC design of these two frameworks have been derived, respectively, and the filter gains were obtained by solving a set of LMIs. A simulation example was provided to demonstrate the effectiveness of the proposed method.

## Author Contributions

All authors listed have made a substantial, direct and intellectual contribution to the work, and approved it for publication.

### Conflict of Interest Statement

The authors declare that the research was conducted in the absence of any commercial or financial relationships that could be construed as a potential conflict of interest.
